# Co-creating a social licence for using novel linked datasets for planning and research in Kent, Surrey and Sussex: results of deliberative focus groups and a creative workshop.

**DOI:** 10.23889/ijpds.v7i3.2109

**Published:** 2022-08-25

**Authors:** Elizabeth Ford, Kathryn Stanley, Melanie Rees-Roberts, Anotida Madzvamuse, Jo Armes, Sarah Giles

**Affiliations:** 1 Brighton and Sussex Medical School; 2 University of Kent; 3 University of Sussex; 4 University of Surrey; 5 Public Contributor

## Objectives

In Kent, Surrey and Sussex (KSS), linked health and social care datasets are in set-up phase in NHS integrated care systems (ICS), and governance models for using data for planning and research are under development. This represented an exceptional opportunity to consult with KSS citizens and work together to identify how ICSs in KSS can secure a social licence for data-linkage and data uses.

## Approach

We held online deliberative discussion focus groups asking KSS citizens to discuss the perceived benefits and risks of data-linkage for planning and research; to describe safeguards they expected around the data, and to describe how the public should be involved in, and communicated with, regarding governance and uses of datasets. We held one creative workshop in which participants artistically depicted their support or concerns around data.

## Results

79 KSS citizens took part in 5 focus groups, and 7 participants attended the creative workshop. There was widespread support for data-linkage to improve efficiency of services and information flows, with the expectation that this would improve patient experience. Proposed ICS governance models were acceptable, but participants identified four key values to ensure appropriate use: acknowledging experience of stigma and discrimination; public voices being heard; holding people to account; and keeping data trails and audits. Participants gave a range of suggestions for ensuring public involvement and communication would be accessible and reach a diverse audience, such as using community champions to ensure a range of contributors, using plain language, giving concise information, building trust through mutually respectful relationships, and valuing public contributions through appropriate payment.

## Conclusion

Social licence theory describes expectations that organisations go beyond requirements of formal regulation and ensure transparent values of reciprocity, non-exploitation and service to the public good. Following findings from this project, ICSs in KSS are now in a good position to deliver social licence values, together with a strong public voice, to inform and determine governance arrangements for linked datasets in the region.

